# Is Iatrogenic Atrial Septal Defect Benign? Rapid Hemodynamic Collapse After M-TEER Redefines a Dynamic Lesion

**DOI:** 10.1016/j.jaccas.2026.108387

**Published:** 2026-06-03

**Authors:** Ryo Horita, Daisuke Hachinohe

**Affiliations:** Department of Cardiovascular Medicine, Asia Medical Group, Sapporo Heart Center, Sapporo Cardio Vascular Clinic, Sapporo, Japan

**Keywords:** iatrogenic atrial septal defect, mitral valve transcatheter edge-to-edge repair

**Figure:**
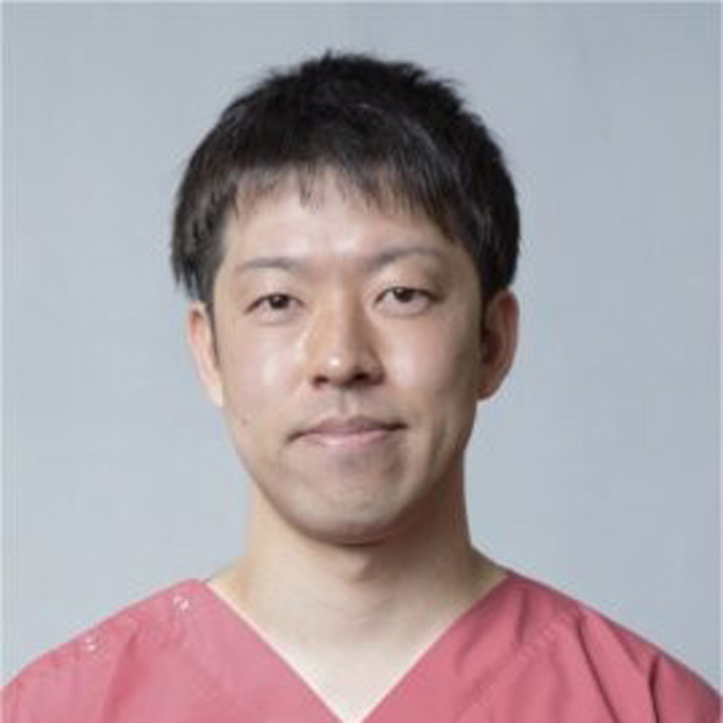

Mitral valve transcatheter edge-to-edge repair (M-TEER) has transformed the management of mitral regurgitation, and iatrogenic atrial septal defect (iASD) has consequently emerged as an unavoidable and frequently encountered finding.^1^ Historically, iASD has been considered “benign.” Although commonly observed in the early postprocedural period, most defects either close spontaneously or remain hemodynamically insignificant.^2^ Furthermore, previous clinical trials have suggested that persistent iASD does not adversely affect long-term outcomes.^3^ In the context of M-TEER, iASD has even been interpreted as potentially beneficial, providing continuous left atrial decompression and functioning similarly to an interatrial shunt device, thereby contributing to the management of left-sided heart failure.

However, a case reported by Takeuchi et al^4^ in this issue of *JACC: Case Reports* challenges this long-standing paradigm. In their report, an initially stable iASD expanded within hours following M-TEER, ultimately leading to rapid hemodynamic collapse.

Importantly, this progression was not the result of gradual structural remodeling. Instead, it was driven by a dynamic mechanical force: a severe tricuspid regurgitation (TR) jet acting as a “hydrodynamic wedge.” Quantitative 3-dimensional transesophageal echocardiography demonstrated progressive expansion of the iASD, clearly indicating force-dependent mechanical disruption.

The first key concept highlighted by this case is that iASD should no longer be viewed as a static anatomical finding but rather as a dynamic lesion whose behavior depends on the surrounding hemodynamic environment. Although iASD is often considered clinically stable,^2^ this case demonstrates that it may undergo rapid morphological change within hours. This observation challenges the conventional understanding of iASD natural history and clinical significance.

Second, this report provides a convincing clinical demonstration of a mechanically vicious cycle. The left-to-right shunt through the iASD increases right ventricular volume load and worsens functional TR. The intensified TR jet increases right atrial pressure and impinges on the atrial septum, further enlarging the iASD. This vicious cycle leads to increasing shunt volume and a simultaneous decrease in cardiac output, ultimately resulting in cardiogenic shock. Importantly, this interaction between shunt flow and structure cannot be captured by static assessment alone.

A critical substrate for this cascade is advanced atrial cardiomyopathy with reduced left atrial compliance. In severe atrial remodeling, the left atrium loses its ability to buffer pressure changes and becomes structurally fragile. The markedly dilated left atrium likely represents a “burnt-out atrium.” Mechanical stress from the TR jet likely concentrated at the iASD, leading to its enlargement.

This case also highlights the importance of afterload mismatch after M-TEER. Reducing severe mitral regurgitation removes a low-resistance pathway for the left ventricle, increasing afterload and revealing underlying dysfunction. This hemodynamic shift may elevate left atrial pressure and worsen right-sided loading conditions, further exacerbating TR and accelerating the pathological cascade.

From a clinical perspective, these findings support a shift in how iASD is evaluated. Assessment should go beyond defect size and include right ventricular function, TR severity, and left atrial function. In patients with advanced atrial remodeling, it may be unsafe to assume that iASD is benign, and early closure should be considered. Recent reviews have also emphasized that no formal guideline-based framework currently exists for iASD assessment and management, and that closure decisions should be guided by patient-specific hemodynamics rather than defect size alone.^1^^,^^5^

This case also highlights important procedural implications. “Stationary deployment” is a key strategy for iASD closure in patients with fragile septal tissue. While balloon sizing and the “wiggle test” are standard in conventional ASD closure, these maneuvers may increase mechanical stress and lead to further tearing of the iASD. Operators should recognize that these steps may not be appropriate in iASD closure and should instead use atraumatic, imaging-guided deployment techniques.

In conclusion, this case shows that iASD is not simply a benign finding but a context-dependent, dynamic lesion. It highlights a “hydrodynamic wedge” mechanism, in which a severe TR jet can mechanically disrupt the atrial septum and drive rapid defect expansion. This process is further amplified in the setting of a “burnt-out” or stiff left atrium, where reduced compliance limits pressure buffering and promotes shunt escalation. Its clinical impact depends on the interaction between hemodynamics and structural vulnerability. These findings call for the reassessment of post–M-TEER risk and provide important insights into the evaluation and management of iASD in current practice.

## Funding Support and Author Disclosures

Drs Horita and Hachinohe are clinical proctors for the MitraClip (Abbot Medical).
